# A study about the relevance of adding acetylsalicylic acid in primary prevention in subjects with type 2 diabetes mellitus: effects on some new emerging biomarkers of cardiovascular risk

**DOI:** 10.1186/s12933-015-0254-8

**Published:** 2015-07-30

**Authors:** Giuseppe Derosa, Amedeo Mugellini, Rosa M Pesce, Angela D’Angelo, Pamela Maffioli

**Affiliations:** Department of Internal Medicine and Therapeutics, University of Pavia, and Fondazione IRCCS Policlinico San Matteo, P.le C. Golgi, 2, 27100 Pavia, Italy; Center for the Study of Endocrine-Metabolic Pathophysiology and Clinical Research, University of Pavia, Pavia, Italy; Laboratory of Molecular Medicine, University of Pavia, Pavia, Italy; PhD School in Experimental Medicine, University of Pavia, Pavia, Italy

**Keywords:** Acetylsalicylic acid, Amlodipine, Cardiovascular risk, Hypertension, New emerging biomarkers, Primary prevention

## Abstract

**Aim:**

To evaluate the relevance of adding acetylsalicylic acid (ASA) in primary prevention in subjects with type 2 diabetes mellitus.

**Methods:**

213 patients with type 2 diabetes mellitus and hypertension were randomized to amlodipine 5 mg, or amlodipine 5 mg + ASA 100 mg for 3 months (Phase A); then, if adequate blood pressure control was reached patients terminated the study; otherwise, amlodipine was up-titrated to 10 mg/day for further 3 months and compared to amlodipine 10 mg + ASA 100 mg (Phase B). We assessed at baseline, at the end of Phase A, and at the end of Phase B the levels of some new emerging biomarkers of cardiovascular risk including: high sensitivity C-reactive protein (Hs-CRP), adiponectin (ADN), tumor necrosis factor-α (TNF-α), interleukin-1β (IL-1β), myeloperoxidase (MPO), soluble CD40 ligand (sCDL40).

**Results:**

Compared to baseline, at the end of Phase A, patients treated with amlodipine 5 mg + ASA 100 mg showed a statistically significant reduction of Hs-CRP (−15.0%), TNF-α (−21.7%), MPO (−9.7%), and sCDL40 (−15.7%), and a statistically significant increase of ADN (+15.0%). These values were significantly better than the ones obtained with amlodipine alone. Similarly, at the end of Phase B, amlodipine 10 mg + ASA significantly lowered Hs-CRP (−18.8%), TNF-α (−15.0%), MPO (−9.2%), and sCDL40 (−20.0%) and increased ADN (+11.8%), with a better effect compared to amlodipine alone.

**Conclusion:**

All biomarkers considered were significantly improved by ASA addition. These data suggest that the use of ASA in primary prevention could be useful in patients with type 2 diabetes mellitus and hypertension.

Trial registration: ClinicalTrials.gov: NCT02064218

## Background

Cardiovascular diseases are the main cause of death, hospitalization and disability among people with type 2 diabetes mellitus [1]. The incidence of cardiovascular disease in people with diabetes is more than double that in people without diabetes, and the mortality rate after a first myocardial infarction is much higher in people with diabetes [2, 3].

For this reason, primary prevention of cardiovascular diseases is very important in people with diabetes. At this regard, the Italian trial MIND-IT (The Multiple Intervention in type 2 Diabetes Italy) showed that a multi-factorial intensive intervention in type 2 diabetes is feasible and effective in clinical practice and it is associated with significant and durable improvement in glycated hemoglobin (HbA_1c_) and cardiovascular disease risk profile [4]. This was confirmed by the Italian guidelines for the treatment of type 2 diabetes mellitus that recommended primary prevention in patients with diabetes throughout changes in lifestyle, glycemic and lipid control, blood pressure control, and possible introduction of anti-platelet therapy [5]. Also the recently published American Diabetes Association guidelines recommend aspirin therapy (75–162 mg/day) as a primary prevention strategy in patients at increased cardiovascular risk, including men aged >50 years or women aged >60 years with, at least, one additional major risk factor (family history of cardiovascular disease, hypertension, smoking, dyslipidemia, or albuminuria) [6]. Despite the higher absolute risk of cardiovascular disease in these patients, however, there is no robust evidence that the use of acetylsalicylic acid (ASA) leads to a favourable benefits-to-risk balance [7]. The JPAD study (Japanese Primary Prevention of Atherosclerosis with aspirin for Diabetes) analyzed the effect of ASA, 81–100 mg, in patients with type 2 diabetes in primary prevention of atherosclerotic events. The risk of cardiovascular disease did not differ between the group receiving ASA and the one that did not, however, among individuals aged 65 years and older, the incidence of atherosclerotic events was significantly lower in the group receiving ASA compared with the group who did not [8]. On the other hand, the POPADAD trial (Prevention of progression of arterial disease and diabetes) trial, conducted in patients with diabetes mellitus and asymptomatic peripheral arterial disease, did not provide evidence to support the use of aspirin in primary prevention of cardiovascular events and mortality in the population with diabetes [9].

Recently some new emerging biomarkers, including soluble CD40 ligand (sCD40L) and serum myeloperoxidase (MPO), have been linked to a higher cardiovascular risk [10]. On this basis the aim of this study was to evaluate the relevance of adding ASA in primary prevention in subjects with type 2 diabetes mellitus. To verify this, we evaluated ASA effects on the levels of some new emerging biomarkers of higher cardiovascular risk in patients with diabetes and hypertension.

## Methods

### Study design

This randomized, double-blind, controlled study was conducted at the Department of Internal Medicine and Therapeutics, University of Pavia, PAVIA, Italy.

The study protocol was conducted in accordance with the Declaration of Helsinki and its amendments, and the Good Clinical Practice Guidelines. It was approved by the each Ethical Committee and all patients provided written informed consent prior to entering the study. Trial registration: ClinicalTrials.gov NCT02064218.

## Patients

We enrolled 213 outpatients (Table 1), aged ≥18 of either sex, satisfying all the following inclusion criteria:

**Table 1:** Baseline characteristics of enrolled patients

Parameters	**N**
N	213
Sex (M/F)	106/107
Age (years)	57.8 ± 7.9
Smokers (M/F)	23/18
BMI (kg/m^2^)	27.7 ± 1.8
HbA_1c_ (%)	6.7 ± 0.7

Data are presented as mean ± standard deviation.

*M* males, *F* females, *BMI* body mass index, *HbA*_*1c*_ glycated hemoglobin.

- overweight (body mass index between 25.0 and 29.9 kg/m^2^);
- mild to moderate hypertension defined by systolic blood pressure (SBP) ≥ 140 mmHg < 180 mmHg and/or diastolic blood pressure (DBP) ≥ 90 mmHg < 105 mmHg;
- normocholesterolemic [low density lipoprotein cholesterol (LDL-C) <160 mg/dl];
- well controlled type 2 diabetes mellitus (HbA_1c_ ≤ 7.5%); all classes of anti-diabetic medications were allowed;
- in primary prevention;
- naïve to anti-hypertensive and anti-platelet treatment in order to avoid possible interactions on primary objective of our study.

The exclusion criteria were secondary hypertension, severe hypertension (SBP ≥ 180 mmHg or DBP ≥ 105 mmHg), hypertrophic cardiomyopathies due to etiologies other than hypertension, history of heart failure, history of angina, stroke, transient ischemic cerebral attack, coronary artery bypass surgery or myocardial infarction any time prior to visit 1, concurrent known symptomatic arrhythmia, liver dysfunction (AST or ALT values exceeding twofold the upper limit), creatinine >1.5 mg/dl, known hypersensitivity to the study drugs. Patients with previous gastric or duodenal bleedings and patients with previous intolerance to ASA were also excluded. Pregnant women as well as women of childbearing potential were excluded.

For all the study duration, no other anti-inflammatory drugs (NSAIDs, immunosuppressive agents, antibiotics, etc.) other than ASA were allowed during the 6 months follow-up.

Suitable subjects, identified from review of case notes and/or computerized clinic registers were contacted personally or by telephone.

### Treatments

**Fig. 1:**
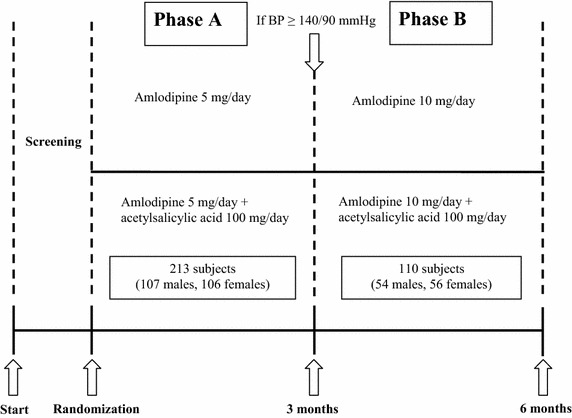
Study design.

The patients fulfilling the inclusion criteria, were randomized to amlodipine 5 mg/day, or amlodipine 5 mg/day + ASA 100 mg for 3 months (Phase A); then, if adequate blood pressure control was reached (BP < 140/90 mmHg), patients terminated the study; otherwise, they proceeded in Phase B of the trial, where amlodipine was up-titrated to 10 mg/day for further 3 months and compared to amlodipine 10 mg + ASA 100 mg (Fig. 1).

All drugs were supplied as identical, opaque, white capsules in coded bottles to ensure the blind status of the study. Randomization was done using a drawing of envelopes containing randomization codes prepared by a statistician. A copy of the code was provided only to the responsible person performing the statistical analysis. The code was only broken after database lock, but could have been broken for individual subjects in cases of an emergency. Medication compliance was assessed by counting the number of pills returned at the time of specified clinic visits. At baseline, we weighed participants and gave them a bottle containing a supply of the study medication for at least 100 days. Throughout the study, we instructed patients to take their first dose of new medication on the day after they were given the study medication. At the same time, all unused medication was retrieved for inventory. All medications were provided free of charge.

### Diet and exercise

All patients were already following a controlled-energy diet (near 600 kcal daily deficit) based on American Heart Association (AHA) recommendations [11] that included 50% of calories from carbohydrates, 30% from fat (6% saturated), and 20% from proteins, with a maximum cholesterol content of 300 mg/day and 35 g/day of fibre. Patients were not treated with vitamins or mineral preparations during the study.

For all the study duration, patients of both arms were encouraged to continue to follow an adequate lifestyle. Standard diet advice was given by a dietician and/or specialist doctor. Dietician and/or specialist doctor periodically provided instruction on dietary intake recording procedures as part of a behaviour modification program and then later used the subject’s food diaries for counselling. Individuals were also encouraged to increase their physical activity by walking briskly for 20–30 min, 3–5 times per week, or by cycling.

### Assessments

Before starting the study, all patients underwent an initial screening assessment that included a medical history, physical examination, vital signs, and a 12-lead electrocardiogram. We assessed blood pressure (BP). We also collected blood sample to evaluate: high sensitivity C-reactive protein (Hs-CRP), adiponectin (ADN), tumor necrosis factor-α (TNF-α), interleukin-1β (IL-1β), MPO, sCDL40. All parameters were assessed at baseline, and after 3 months (at the end of Phase A) for all patients, and after further 3 months (at the end of Phase B only) only for patients proceeding in Phase B of the trial.

All plasmatic parameters were determined after a 12-h overnight fast. Venous blood samples were taken for all patients between 08.00 and 09.00 A.M. We used plasma obtained by addition of Na_2_-EDTA, 1 mg/ml, and centrifuged at 3,000*g* for 15 min at 4°C. Immediately after centrifugation, the plasma samples were frozen and stored at −80°C for no more than 3 months. All measurements were performed in a central laboratory.

Blood pressure measurements were obtained from each patient (left arm) in the sitting position by physicians blinded to treatment using a standard mercury sphygmomanometer (Erkameter 3000; ERKA, Bad Tolz, Germany) (Korotkoff I and V) with a cuff of appropriate size. Blood pressure has been always measured in the morning before daily drug intake (i.e., at trough 22–24 h after dosing) and after the subject has rested 10 min in a quiet room. Three successive BP readings were obtained at 1-min intervals and averaged.

Heart rate was measured by pulse palpation for 30 s, just before the BP measurements.

Body weight was measured with light clothes and without shoes and BMI was calculated as the weight in kg divided by height in m squared.

High sensitivity C-reactive protein was measured with use of latex-enhanced immunonephelometric assays on a BN II analyser (Dade Behring, Newark, Delaware, USA). The intra- and interassay coefficient of variations (CsV) were 5.7 and 1.3%, respectively [12].

Adiponectin level was determined using Enzyme-Linked Immunosorbent Assay (ELISA) kits (B-bridge International, Sunnyvale, CA, USA). Intraassay CsV were 3.6% for low-control sample and 3.3% for high-control sample, whereas interassay CsV were 3.2% for low-control sample and 7.3% for high-control samples, respectively [13].

Tumor necrosis factor-α level was assessed using commercially available ELISA kits according to manufacturer’s instructions (Titer-Zyme EIA kit; Assay Designs, Ann Arbor, MI, USA). Intraassay CsV were 4.5% for low- and 3.6% for high-concentration samples, whereas the interassay CsV were 6.0% for low and 11.8% for high-concentration samples, respectively [14].

We used a human cytokine 27-Bio-Plex assay kit (BioRad Laboratories, Milan, Italy), a bead-based multiplex immunoassay for IL-1β. This technology has the capacity to measure several cytokines/cytokine receptors and growth factors simultaneously in small volumes of plasma with high accuracy and sensitivity [15]. The lower detection limit was 0.2–19.3 pg/mL. The samples were read on a Bio-Plex 200 instrument equipped with the software bioplex manager, version 4.1, (Bio-Rad Laboratories, Hercules, CA, USA), using a five-parameter non-linear regression formula to compute sample concentrations from the standard curves.

Myeloperoxidase was assessed using commercially available ELISA kits according to manufacturer’s instructions (R & D Systems, Minneapolis, MN, USA). The intra- and interassay CsV were 7.7 and 8.3%, respectively [16].

Soluble CD40 ligand was assessed using commercially available ELISA kits according to manufacturer’s instructions (R & D Systems, Minneapolis, MN, USA). The intra- and interassay CsV were 4.5 and 6.0%, respectively [17].

### Statistical analysis

Data are expressed as mean ± standard deviation (SD). The statistical analysis of the data was performed by the statistical analysis software (SAS) system, version 6.12 (SAS Institute, Inc., Cary, NC, USA). The differences between the two groups in baseline characteristics were analyzed by the two-tailed Student’s *t* test. Intervention effects were adjusted for additional potential confounders (sex, smoking status, and age) using analysis of covariance (ANCOVA). Continuous variables were tested using a two-way repeated measures analysis of variance (ANOVA). Differences between baseline and 3-months of treatment in each group were analyzed with the Wilcoxon signed rank test [18]. Non-parametric tests were also employed in the statistical analysis of the data, because some data were not normally distributed (Kolmogorov–Smirnov test). The statistical significance of the independent effects of treatments on the other variables was determined using ANCOVA taking the baseline level of each parameter as a covariate.

Findings of p < 0.05 were considered significant. Considering as clinically significant a difference of at least 10% compared with the baseline and an alpha error of 0.05, the actual sample size was adequate to obtain a power higher than 0.80 for all measured variables.

## Results

### Study sample

We enrolled 213 patients; 107 were randomized to amlodipine 5 mg, and 106 to amlodipine 5 mg + ASA 100 mg; 110 patients did not reach an adequate blood pressure control and continued in the second phase of the study with up-titration to amlodipine 10 mg (54 subjects) or amlodipine 10 mg + ASA 100 mg (56 subjects). Five patients did not complete the first phase of the study (four patients in amlodipine 5 mg group and one patient in amlodipine 5 mg + ASA group) and three patients (two patients in amlodipine 10 mg group and one patient in amlodipine 10 mg + ASA group) did not complete the second phase of the trial. The reason for premature withdrawal were: lost to follow-up, peripheral edema, epigastralgy, withdrawal of informed consent. At baseline, no differences between the two groups were recorded. A list of anti-diabetic treatments taken at the beginning of the study was reported in Table 2.

**Table 2:** Anti-diabetic drugs taken before randomisation

Parameters	**N**
N	213
M/F	106/107
Lifestyle	5 (2.34) 2/3)
Sulfonylureas, n (%) (M/F)	42 (19.7) (18/24)
Glyburide	3 (7.1) (1/2)
Glimepiride	17 (40.5) (10/7)
Gliclazide	22 (52.4) (12/10)
Biguanides, n (%) (M/F)	153 (71.8) (71/82)
Metformin	153 (100) (71/82)
Glinides, n (%) (M/F)	32 (15.0) (17/15)
Repaglinide	32 (100) (17/15)
α-glucosidase inhibitors, n (%) (M/F)	33 (15.5) (14/19)
Acarbose	33 (100) (14/19)
Thiazolidinediones, n (%) (M/F)	28 (13.1) (15/13)
Pioglitazone	24 (85.7) (12/12)
Rosiglitazone	4 (14.3) (3/1)
DPP-4 inhibitors, n (%) (M/F)	33 (15.5) (17/16)
Sitagliptin	12 (36.4) (6/6)
Vildagliptin	10 (30.3) (6/4)
Saxagliptin	7 (21.2) (3/4)
Linagliptin	4 (12.1) (2/2)
GLP-1 analogs, n (%) (M/F)	15 (7.0) (8/7)
Exenatide	10 (66.7) (7/3)
Liraglutide	5 (33.3) (2/3)

*M* males, *F* females, *DPP-4* dipeptidyl peptidase-4, *GLP-1* glucagon-like peptide-1.

### Blood pressure

We recorded a decrease of SBP (−6.5% with amlodipine 5 mg, −5.9% with amlodipine 5 mg + ASA, −13.2% with amlodipine 10 mg, −13.5% with amlodipine 10 mg + ASA). A similar trend was observed for DBP (−8.2% with amlodipine 5 mg, −9.4% with amlodipine 5 mg + ASA, −14.8% with amlodipine 10 mg, −15.1% with amlodipine 10 mg + ASA). No differences between amlodipine or amlodipine + ASA were recorded (Tables 3, 4).

**Table 3:** Data of patients completing Phase A of the trial

Parameters	Amlodipine 5 mg		Amlodipine 5 mg + ASA 100 mg	
	Baseline	3 months	Baseline	3 months
N	107	103	106	105
Sex (M/F)	54/53	51/52	53/53	53/52
Smokers (M/F)	12/10	11/10	12/9	12/8
SBP (mmHg)	156.4 ± 9.4	145.2 ± 8.4*	153.6 ± 9.0	146.1 ± 8.8*
DPB (mmHg)	95.9 ± 5.5	88.9 ± 4.1*	97.2 ± 5.9	87.7 ± 3.9*
Hs-CRP (mg/l)	2.1 ± 0.9	1.9 ± 0.7	1.9 ± 0.7	1.7 ± 0.5*^
ADN (μg/ml)	5.4 ± 1.2	5.6 ± 1.2	5.2 ± 1.0	6.2 ± 1.8*^
TNF-α (pg/ml)	2.4 ± 0.9	2.1 ± 0.7	2.3 ± 0.8	1.8 ± 0.5*^
IL-1β (pg/ml)	0.6 ± 0.5	0.5 ± 0.3	0.6 ± 0.5	0.3 ± 0.2*
MPO (ng/ml)	780.1 ± 220.3	740.3 ± 220.4	776.5 ± 218.5	702.7 ± 114.4*^
sCDL40 (pg/ml)	1254.4 ± 110.2	1231.3 ± 102.1	1260.7 ± 119.5	1064.5 ± 92.9*^

Data are expressed as mean ± standard deviation.

*M* males, *F* females, *Hs-CRP* high sensitivity C-reactive protein, *ADN* adiponectin, *TNF-α* tumor necrosis factor-α, *IL-1β* interleukin-1β, *MPO* myeloperoxidase, *sCDL40* soluble CD40 ligand.

* p < 0.05 vs baseline.

^ p < 0.05 vs amlodipine.

**Table 4:** Data of patients entering the Phase B of the trial

Parameters	Amlodipine 10 mg		Amlodipine 10 mg + ASA 100 mg	
	Baseline	3 months	Baseline	3 months
N	54	52	56	55
Sex (M/F)	25/29	24/28	29/27	28/27
Smokers (M/F)	4/3	4/3	5/3	5/3
SBP (mmHg)	153.7 ± 9.1	134.8 ± 6.1°	154.2 ± 9.2	134.2 ± 5.9°
DPB (mmHg)	95.1 ± 5.2	82.5 ± 3.2°	96.8 ± 5.7	82.2 ± 3.1°
Hs-CRP (mg/l)	2.0 ± 0.8	1.6 ± 0.5*	2.0 ± 0.8	1.3 ± 0.4°^
ADN (μg/ml)	5.1 ± 1.1	6.0 ± 1.5*	5.3 ± 1.2	6.8 ± 1.9°^
TNF-α (pg/ml)	2.5 ± 0.8	2.0 ± 0.6*	2.2 ± 0.7	1.7 ± 0.4°^
IL-1β (pg/ml)	0.5 ± 0.8	0.5 ± 0.3	0.5 ± 0.4	0.3 ± 0.2*
MPO (ng/ml)	775.2 ± 219.5	728.1 ± 217.3*	774.2 ± 216.2	661.2 ± 112.3°^
sCDL40 (pg/ml)	1252.8 ± 118.9	1198.4 ± 99.7*	1268.5 ± 118.2	958.3 ± 82.4°^

Data are expressed as mean ± standard deviation.

*M* males, *F* females, *Hs-CRP* high sensitivity C-reactive protein, *ADN* adiponectin, *TNF-α* tumor necrosis factor-α, *IL-1β* interleukin-1β, *MPO* myeloperoxidase, *sCDL40* soluble CD40 ligand.

* p < 0.05 vs baseline.

° p < 0.01 vs baseline.

^ p < 0.05 vs amlodipine.

### New markers of cardiovascular risk

After 3 months of therapy, no variations of the above cited markers were recorded with amlodipine alone. Patients treated with amlodipine 5 mg + ASA 100 mg, instead, showed a reduction of MPO, and sCDL40, compared to baseline (−9.7 and −15.7%, respectively), and to amlodipine alone (−5.1 and −13.6%). One hundred and seven patients continued the study, and were up-titrated to amlodipine 10 mg + ASA 100 mg or to amlodipine 10 mg alone. We observed a decrease of MPO (−6.4% for amlodipine alone, and −15.0% for amlodipine + ASA), and sCDL40 (−5.1% for amlodipine alone, and −24.1% for amlodipine + ASA) in both groups compared to baseline, even if values recorded with amlodipine 10 mg + ASA were lower than the ones recorded with amlodipine 10 mg alone (−9.2 and −20.0%, respectively) (Tables 3, 4).

### Inflammatory markers

We did not record any variations of inflammatory markers after 3 months of amlodipine monotherapy. In the group treated with amlodipine 5 mg + ASA 100 mg, instead, there was a reduction of Hs-CRP (−15.0%), and TNF-α (−21.7%), and an increase of ADN (+15.0%) compared to baseline, and to amlodipine alone (−10.5, −14.3 and +9.7%, respectively). Regarding IL-1β, it decreased with amlodipine 5 mg + ASA 100 mg compared to baseline (−50.0%), but no differences were recorded compared to amlodipine alone. In patients continuing the study, we recorded a decrease of Hs-CRP (−20.0% with amlodipine and −35.0% with amlodipine +ASA), and TNF-α (−13.1% with amlodipine and −26.1% with amlodipine + ASA), and an increase of ADN (+11.7% with amlodipine and +22.1% with amlodipine + ASA) compared to baseline. Values recorded with amlodipine 10 mg + ASA were better than the ones recorded with amlodipine 10 mg alone (−18.8, −15 and +11.8%, respectively). Regarding IL-1β, it decreased compared to baseline only with amlodipine 10 mg + ASA (−50%) (Tables 3, 4).

### Adverse events

No significant serious adverse events were reported. We recorded six episode of epistaxis, and four episodes of epigastralgy in patients taking ASA, and four episodes of peripheral edema in amlodipine 10 mg groups; all events were reported as mild.

## Discussion

We observed that the addition of ASA to amlodipine therapy in patients with diabetes was effective, in primary prevention, in reducing some inflammatory and new emerging biomarkers in cardiovascular risk stratification, suggesting a favourable effects of ASA in this kind of patients. In particular, our data showed a reduction of Hs-CRP not reported by Vaucher et al. [19]. These Authors reported that low-dose aspirin for cardiovascular prevention does not impact plasma pro-inflammatory cytokines and Hs-CRP levels, however, in their study, only a small portion of the studied population was affected by diabetes, while our population was all affected by diabetes.

In our study we also observed a reduction of MPO and sCDL40 in patients treated with ASA. Reduction of sCDL40 was reported also by Rosiak et al. that reported a significant reduction of Hs-CRP, sCD40L, and interleukin-6 with ASA [20].

Differently from what reported in literature [21, 22] where, in patients with diabetes, age proved to be the most important predictive factor of laboratory response to ASA therapy, we did not record different effects of ASA according to different age; this is probably due to the fact that, in our population, age standard deviation was very low, suggesting a homogeneous data. The anti-inflammatory action observed in our study can be only partially explained by blood pressure reduction, because amlodipine dose was identical in both arms. Moreover, we chose to use amlodipine as anti-hypertensive agent because, from the evidence published in literature, amlodipine proved to be neutral on Hs-CRP, both used alone [23], or in addition to atorvastatin [24], even if it increased adiponectin both in combination with atorvastatin [24] and olmesartan [25]. All data collected suggest a protective effect linked to ASA use in primary prevention in patients with diabetes.

Regarding the mechanism of action throughout ASA acts, it is largely known that ASA is the archetypal non steroidal anti-inflammatory drug found to inhibit the cyclooxygenase (COX II) pathway of arachidonic acid metabolism [26], being anti-inflammatory at 1 g dose [27], but cardioprotective at lower doses (75–150 mg/day) through the inhibition of platelet-derived thromboxane (Tx) A_2_ [28, 29]. ASA also inhibits pathways inherent to innate immunity including the production of TxA_2_ [30], which is suggested to facilitate the polymorphonuclear leukocyte (PMN)-platelet interaction that leads to PMN transmigration into inflamed tissues [31]. Moreover, ASA triggers the synthesis of novel lipid metabolites that directly halt leukocyte trafficking and elicit pro-resolution effects [32]. In addition, there is evidence that ASA down-regulates pro-inflammatory signaling pathways including NF-κB [33]. This suggests that ASA may be anti-inflammatory at levels used in cardio-protection. This was confirmed by Morris et al. [34] that showed that ASA dampens innate immuno-mediated responses in humans by triggering 15-epi-lipoxin A4 from endothelial COX2 expressed in response to local injury, which subsequently prevents leukocyte accumulation to sites of tissue injury in an NO-dependent manner.

Of course our study has some limitations: for example, we evaluated only some inflammatory parameters and some new markers of cardiovascular disease, focusing our attention on a few of them. Moreover, it would be interesting to verify if the reduction of these biomarkers will have an impact on the reduction of cardiovascular events, but, to assess this, longer studies are needed.

## Conclusions

The addition of ASA to amlodipine gave a better improvement of inflammatory parameters compared to amlodipine alone, suggesting a role of ASA in reducing inflammation and endothelial damage independently from the blood pressure reduction. These data suggest that the use of ASA in primary prevention could be useful in patients with type 2 diabetes mellitus and hypertension.
